# Research on the Early-Age Cracking of Concrete Added with Magnesium Oxide under a Temperature Stress Test Machine

**DOI:** 10.3390/ma17010194

**Published:** 2023-12-29

**Authors:** Zixuan Li, Zheng Chen, Junting Li, Zhiwen Xu, Weilun Wang

**Affiliations:** College of Civil and Transportation Engineering, Shenzhen University, Shenzhen 518060, China; lizixuan2021@email.szu.edu.cn (Z.L.); chenzheng2020@email.szu.edu.cn (Z.C.); lijunting2017@email.szu.edu.cn (J.L.); xuzhiwen2021@email.szu.edu.cn (Z.X.)

**Keywords:** early deformation, restraining stress, stress relaxation, temperature stress test

## Abstract

Concrete cracking is a significant issue in the global construction industry, and the restraint stress of concrete is a crucial contributing factor to early concrete cracking. The addition of magnesium oxide additive (MEA) to concrete is a method to enhance its crack resistance. In this paper, concrete specimens with four different contents of MEA were tested with a temperature stress testing machine. The deformation characteristics and mechanical properties of concrete with varying contents of MEA were investigated using both free deformation tests and fully constrained deformation tests. The prediction model for the early restrained stress of concrete was developed by integrating the stress relaxation phenomenon of concrete with models for autogenous shrinkage, temperature deformation, and elastic modulus. According to the results, (1) the thermal expansion coefficient exhibits a pattern of initially increasing and subsequently decreasing with the increasing ratio of MEA; (2) the addition of 3% and 8% MEA can offset 23% and 35.1% of the concrete’s self-shrinkage, respectively. Nevertheless, when the added MEA content is 5%, the self-shrinkage of concrete increases by 6%; (3) the addition of 3–8% MEA can result in a 0.5–1.67 times increase in the maximum expansion stress of concrete, as well as a 0.5–0.95 times increase in cracking stress; (4) as the MEA content continues to increase, the stress relaxation level of concrete also increases. In comparison to concrete mixed without MEA, the maximum increase in the stress relaxation level of concrete is 65.5%, thereby enhancing the concrete’s anti-cracking ability. However, when the MEA dosage reaches a certain threshold, the stress relaxation enhancement brought about by the addition of MEA will no longer be significant; (5) when compared to the experimental data, the established model of early-age constraint stress accurately predicts the tensile constraint stress of concrete.

## 1. Introduction

Concrete cracking is a complex and challenging problem that represents a significant hurdle in the field of engineering. Concrete cracking not only impacts a building’s appearance but also facilitates the ingress of harmful substances like chloride ions into the structure, leading to steel bar corrosion and diminished building durability [1].

The early deformation of concrete results in constraint stress and is one of the primary factors contributing to early concrete cracking [2]. Autogenous shrinkage and temperature deformation are the key manifestations of early deformation in concrete [3]. Lyman [4] discovered and documented the phenomenon of autogenous shrinkage in 1934. This is a combined effect of chemical shrinkage and self-desiccation during concrete hydration. Temperature deformation occurs in concrete due to temperature changes, and the thermal expansion coefficient of concrete is the key parameter governing this transformation. Currently, numerous scholars have developed mathematical models to predict the early-age development of the thermal expansion coefficient in concrete. Lin Zhi Hai [5] revealed that the thermal expansion coefficient of concrete at an early age is closely related to the hardening process of concrete, and Wei Ding [6] proposed a piecewise development expression of the thermal expansion coefficients of cement paste and mortar at an early age through the study of cement paste, mortar, and concrete.

The elastic modulus and stress relaxation are inherent properties of concrete and play significant roles in influencing early cracking. The elastic modulus, which largely depends on the paste and aggregate of concrete, is a crucial parameter for calculating deformation and crack propagation. Numerous scholars have proposed more reliable empirical formulas for calculating the elastic modulus [7,8]. Stress relaxation is a phenomenon resulting from the creep properties of concrete. It involves a gradual decrease in stress over time while maintaining constant total deformation. Early-age stress relaxation in concrete can effectively alleviate restraint stress and delay the occurrence of early cracking.

Moreover, the inclusion of a concrete expansion agent will also impact the formation of cracks in concrete. Magnesium oxide addictive (MEA) finds extensive application in hydraulic engineering projects due to its ability to achieve complete early-stage reactivity and provide excellent expansion stability in the later stages, given suitable activity levels and dosages. An examination of the MEA’s long-term application history reveals its capacity for inducing expansion strain, enabling it to counteract the shrinkage strain in concrete and subsequently mitigate the risk of cracking [9,10,11].

Various and complex factors affect concrete cracking, making it difficult to conduct a comprehensive quantitative study with only the standard methods using length comparators [12] and dual rings [13]. The temperature stress test is a method used to study the crack resistance of concrete, employing a temperature stress testing machine (TSTM). The temperature stress test can generate more quantitative data than the methods using length comparators and dual rings. These data allow for the measurement of concrete deformation, restraint stress, and elastic modulus, providing a reliable assessment of concrete crack resistance.

Recently, numerous research teams have utilized TSTM to investigate the crack resistance of concrete. Wang [14] conducted a systematic study on the impact of three different types and three varying amounts of MgO on the shrinkage and crack resistance of panel concrete using slab tests, restrained drying shrinkage tests, and TSTM. The findings suggest that the proper addition of MEA can enhance the compressive and tensile strength of concrete, as well as significantly improve its elastic modulus, permeability, and crack resistance. Through temperature stress tests, Xianfeng Wang [15] and his research team found that seawater and sea sand lead to increased early self-shrinkage of concrete, in comparison to ordinary concrete. Using a novel approach with the self-developed TSTM, Guangfeng Ou [16] evaluated the early-age cracking sensitivity of concretes blended with calcium sulfoaluminate and MgO expansive additives under different engineering conditions.

Currently, research on the impact of MEA on concrete cracking mainly falls into two categories. One type of research examines the combined effect of MEA and other admixtures on concrete, while the second type investigates the long-term cracking of MEA in concrete [17,18,19]. Nevertheless, research on the impact of MEA addition on the early-age cracking of concrete is still inadequate. This article involves the analysis of the impact of MEA, a single variable, on the working and mechanical properties of concrete by configuring different amounts of MEA in the concrete. In addition, a temperature stress testing machine is utilized to conduct experimental research on the early deformation and stress characteristics of externally mixed MEA concrete. Moreover, a corresponding mathematical model is established to predict its early-age stress and impact, instead of relying solely on on-site experience. This research aims to provide a reliable reference for future research aiming to improve the crack resistance performance of concrete.

## 2. Methods and Materials

### 2.1. Materials

For the temperature stress test, two repeated concrete specimens were prepared for each experimental group and control group to conduct both fully restrained and free deformation tests.

The raw materials used for preparing the specimens were as follows:

(1) Cement

The cement used in this study is PO42.5 cement, which conforms to the specifications of the general Portland cement standard (GB175-2007) [20]. The diverse properties and composition of the cement are presented in Table 1.

(2) Coarse and fine aggregates

Two different gradations of basalt, 5–10 mm and 10–20 mm, were used in this experiment, mixed in a 1:2 mass ratio. The coarse aggregate part’s screening table is presented in Table 2. The fine aggregate has a maximum sand particle size of 5 mm and a fineness modulus of 2.4.

(3) Magnesium oxide expansion agent

The magnesium oxide expansion agent (MEA) utilized in this experiment complies with the technical specifications outlined in “Concrete Expansion Agent” (GB/T 23439-2017) [21], and its diverse properties are presented in Table 3.

(4) Mix proportion of concrete

The specimens in this experiment were prepared using the absolute volume method recommended by JGJ/T 283-2012 [22] “Technical Specification for Self-compacting Concrete application”. Once the mix proportion for the control group was determined, MEA was added in varying proportions based on the quality of the cementitious materials. The final mix proportion is presented in Table 4.

The letter “H” in the identifier denotes self-compacting concrete, while “HM” signifies self-compacting concrete with added MEA. The numerical portion of the identifier, such as “HM-1”, corresponds to MEA with a mass fraction of 3% of cementitious material, introduced through the external mixing method in the mix proportion. “HM-2” and “HM-3” correspond to MEA with mass fractions of 5% and 8% added using the external mixing method, respectively.

### 2.2. Experimental Methods

#### 2.2.1. Specimen Preparation

The specimen had a dog-bone shape similar to that of the axial tensile test, with an effective length of 1200 mm and a section size of 120 × 120 (mm).

The specific preparation process was as follows:

1. The insulation cover plate was removed from the upper part of the TSTM, and a double layer of plastic film was added to the concrete mold. Prior to applying the film, insulation panels were positioned on the three surfaces that came into contact with the concrete within the effective length of the mold to minimize temperature errors resulting from the high thermal conductivity of the metal.
2. The mixed concrete was poured into a concrete test chamber with attached plastic film, ensuring that the pouring height was 0.5 cm from the top end face of the concrete clamp mold. Based on the concrete’s fluidity, two sections of ribbed steel bars were positioned at the stress concentration points at both ends at the appropriate times to prevent stress concentration and premature fracture during the testing process.
3. After the concrete was poured, a layer of cling film was applied to the concrete surface, ensuring that it was tightly adhered to prevent moisture loss and isolate drying shrinkage. At this stage, the preparation of concrete specimens was typically completed.

#### 2.2.2. The Experimental Equipment

The first TSTM [23] was invented to study the susceptibility of concrete to early cracking, allowing for precise control over restraint and achieving a 100% restraint effect. Over time, the accuracy of recording devices has greatly improved, and the scope of the application of TSTM has significantly expanded through continuous upgrading. The machine has recently been used to study various important properties of concrete, including autogenous shrinkage [24], the coefficient of thermal expansion [25], creep relaxation [26,27,28,29,30,31,32,33], and tensile strength [34], as well as the susceptibility to early cracking.

**Figure 1:**
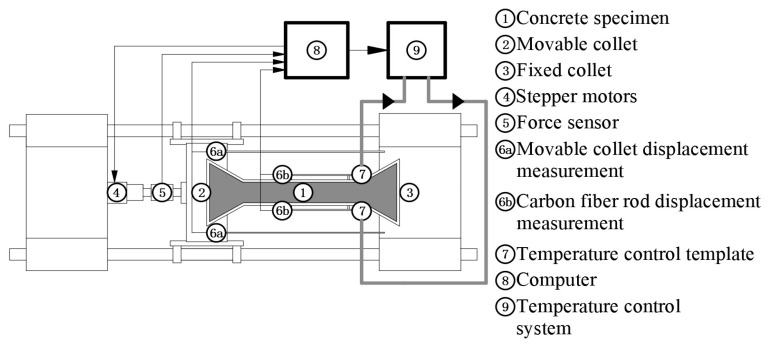
Structure diagram of temperature stress testing machine.

As shown in Figure 1, the test was performed using a novel TSTM developed by Shenzhen Concrete Source Equipment Company Shenzhen, China, which is composed of a mechanical frame and a temperature control system, with a displacement control accuracy of 1 μm. The measurement accuracy was 0.1 μm.

#### 2.2.3. Working Mode of TSTM

There are two main working modes for TSTM: fully constrained mode and free deformation mode.

Measurement of deformation in free deformation mode: During the test, concrete was poured into the concrete curing box of the equipment. The TSTM consists of concrete collet dies, with one end being the fixed end and the other end being the active end. During the free deformation measurement of concrete, the concrete undergoes expansion or contraction due to the hydration reaction. This creates pressure or tension on the concrete collet. When the stress measurement value of the load sensor reaches the set threshold, the servo motor activates, causing the displacement of the active end. The stress induced by deformation is released to restore the constraint stress to zero. The fixed displacement vertical rod on the clamp die is connected to the linear variable differential transformer (LVDT). The relative displacement of the rod can be used to measure the deformation of the concrete.

Measurement of stress in a fully constrained mode: In this mode, the threshold for the constrained displacement can be set based on the test requirements. When the expansion or contraction value reaches the set threshold, the servo motor is activated to forcibly restore the concrete to its original length, and the stress of the concrete is calculated in real time using the measured value from the force sensor.

#### 2.2.4. Test Method

For the experimental process, we referred to previous studies on the early performance of concrete using TSTM [35,36] and the study by TSTM developers [5]. The specific testing process of TSTM was as follows:

1. After selecting the operating mode (free deformation mode or fully constrained mode), the TSTM was initiated, the internal core temperature of the specimen was monitored, a temperature control system was employed to maintain the concrete specimen in a semi-adiabatic state, and the synchronous motor was simultaneously activated to ensure that the specimen was in a 100% constrained state.
2. Once the internal temperature of the concrete specimen reached its peak (i.e., the internal core temperature stabilized), the temperature control system was turned off, and the concrete specimen was allowed to cool naturally until it developed cracks.
3. Once the specimen cracked, the test was concluded, and the observational data were collected for analysis.

**Figure 2:**
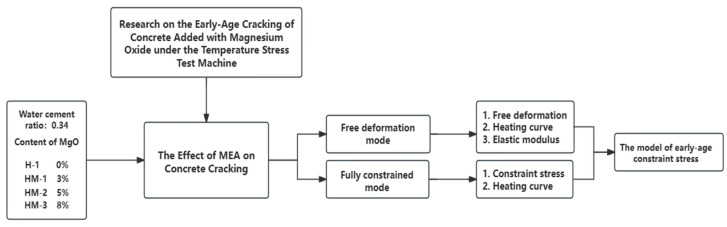
The research flowchart of this article.

The research flowchart of this article is presented in Figure 2.

## 3. Results and Discussion

Several studies have demonstrated that the external addition of MEA significantly affects the mechanical properties and cracking of concrete.

Lei Wang and colleagues [14] demonstrated that the proper addition of MEA effectively enhances the compressive and tensile strength of concrete while significantly improving its elastic modulus, permeability, and crack resistance.

Research by Shunkai Li [37] revealed that as the MEA content increases, the autogenous shrinkage of concrete gradually decreases. However, excessively high MEA content may diminish the strength and durability of concrete. Additionally, when the water–cement ratio of concrete is low, the influence of MEA content on concrete-cracking sensitivity becomes more pronounced.

Xia Chen, Hua Quan Yang [38], and their colleagues investigated various dosages of lightly burned MEA concrete to examine influencing factors such as calcination conditions, batching methods, and raw material changes. Their comparison of cracking sensitivity among different concrete proportions demonstrated the beneficial impact of MgO on enhancing concrete’s crack resistance.

### 3.1. Effect of MEA on the Expansion Coefficient of Concrete

The temperature strain and temperature change value of concrete were measured using TSTM in the free deformation mode, and by utilizing the thermal expansion coefficient calculation model developed by Zhang Tao [39] from Tsinghua University, the thermal expansion coefficient of each group of specimens was calculated, as shown in Table 2.

Zhang Tao’s thermal expansion coefficient calculation model is as follows:

(1) $$\alpha_{T}\left(t\right)=\alpha_{t}\times \left(1+41\times t^{-m}\right)$$

(2) $$\alpha_{t}=\frac{∆\varepsilon }{∆T}$$

(3) $$\varepsilon_{th}\left(t\right)=\sum \alpha_{T}\left(t\right)\cdot ∆T$$

In the above formula, $\alpha_{T}\left(t\right)$ is the thermal expansion coefficient of concrete at time *t*; *t* is the age of the concrete, and the unit is h; *m* is the development coefficient of the thermal expansion coefficient, and the value is 2; ∆ε is the temperature strain of the concrete, and the unit is με; ∆T is the temperature change value of concrete, and the unit is °C; and $\varepsilon_{th}\left(t\right)$ is the temperature deformation of concrete.

The measurement results in Table 5 show that the thermal expansion coefficient initially increases and then decreases with the increase of MEA doping rate. According to the research conducted by Gui Bo Gao [40] and other teams, it is known that the coefficient of thermal expansion is related to the density of the material, and materials with a compact and dense structure tend to have higher coefficients of thermal expansion. In mixtures with lower initial content, the delayed hydration property of MgO contributes to filling the cracks in the cement particles, resulting in a denser cement structure and improving the coefficient of thermal expansion to some extent. However, as the dosage continues to increase, an excess of MgO particles filling the cracks in the cement paste gradually appears, resulting in a certain decrease in the density of the cement slurry during the initial stage of hydration. In addition, the hydration reaction of MgO particles during concrete curing leads to the formation of Mg(OH)_2_ crystals, resulting in expansion and extrusion inside the concrete. In some weaker areas, this process can even cause the formation of microcracks, leading to increased porosity and a gradual decrease in the thermal expansion coefficient of the concrete.

**Figure 3:**
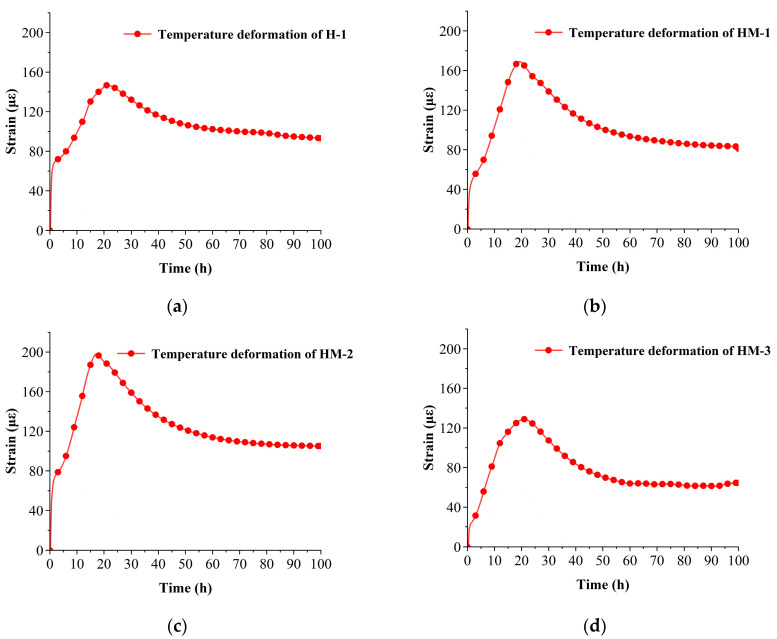
The temperature deformation of each group of specimens: (**a**) the temperature deformation curve of H-1; (**b**) the temperature deformation curve of HM-1; (**c**) the temperature deformation curve of HM-2; (**d**) the temperature deformation curve of HM-3.

According to Formula (3), the temperature deformation curves of each group of specimens were plotted, and the results are depicted in Figure 3. A comparison of the temperature deformation curves of each specimen group reveals that an increase in MEA content alters the thermal expansion coefficient, leading to a trend of initial increase and subsequent decrease in the maximum temperature deformation of the concrete specimen, which is equivalent to the thermal expansion coefficient.

### 3.2. Effect of MEA on the Autogenous Shrinkage of Concrete

The deformation of the specimen in the temperature stress test primarily consists of autogenous shrinkage and temperature deformation. If the focus of the study is on the autogenous shrinkage of concrete, the temperature deformation can be separated from the total deformation.

**Figure 4:**
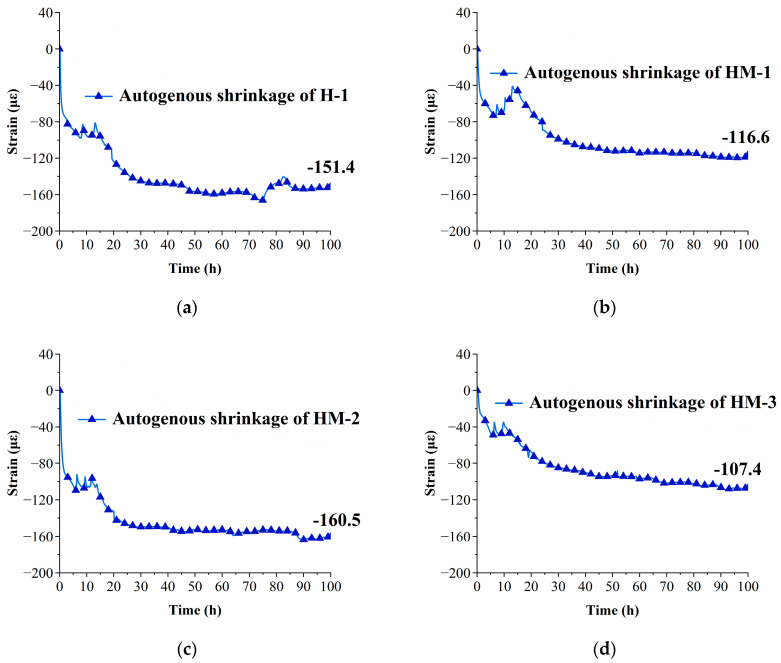
The autogenous shrinkage curve of each group: (**a**) the autogenous shrinkage curve of H-1; (**b**) the autogenous shrinkage curve of HM-1; (**c**) the autogenous shrinkage curve of HM-2; (**d**) the autogenous shrinkage curve of HM-3.

After deformation separation, the autogenous shrinkage curve of each group was drawn, and the results are shown in Figure 4.

The figure clearly demonstrates a significant reduction in the autogenous shrinkage of concrete after the addition of MEA. The autogenous shrinkage values of the H-1, HM-1, HM-2, and HM-3 specimens at 100 h are −151.4, −116.6, −160.5, and −107.4, respectively. When comparing the autogenous shrinkage values of each concrete group at 100 H, it is observed that the values of autogenous shrinkage decrease by 23% and 35.1% with MEA contents of 3% and 8%, respectively. However, a 6% increase in autogenous shrinkage value is observed when the MEA content is 5%.

Autogenous shrinkage exhibits a trend of an initial decrease, followed by an increase, and a final decrease. This is because when a small amount of MEA is added, the water in the concrete paste is more sufficient, which promotes the smooth hydration of most MgO particles and the formation of Mg(OH)_2_ crystals. These crystals contribute to the reduction in autogenous shrinkage. However, with a further increase in dosage, the excessive addition of MEA results in insufficient water for the normal hydration of cementitious materials [14]. Consequently, cement stone and Mg(OH)_2_ crystals are not sufficiently produced, leading to the formation of numerous micropores within the microstructure. This phenomenon contributes to the significant occurrence of autogenous shrinkage. As the amount of MEA continues to increase, although the hydration of a substantial number of MgO crystals during the early hydration stage is insufficient to form a sufficient amount of Mg(OH)_2_ crystals, the addition of numerous MgO crystals with smaller crystal sizes than cement particles and cement stone contributes to the filling of microcracks and the reduction in capillary pressure. Hence, it further diminishes the magnitude of autogenous shrinkage [37,41].

### 3.3. Effect of MEA on the Restraint Stress of Concrete

In the fully constrained mode of the TSTM, the equipment’s servo motor consistently limits the deformation of the concrete specimen to 1 μm, ensuring 100% restraint stress in the concrete specimen. In this mode, the equipment’s force sensor can measure the restraint stress exerted by the concrete specimen.

The peak expansion stress and cracking stress of the concrete increase gradually with the increasing MEA content, as revealed by the results presented in Table 6. This observation can be primarily attributed to the following factors: (1) The addition of MEA leads to a reduction in the water–binder ratio of the concrete. A lower water–binder ratio results in higher peak expansion stress and cracking stress in the concrete [42]; and (2) MEA undergoes hydration within the microcracks, forming Mg(OH)_2_ crystals, which fill the micropores in the concrete, thereby enhancing its cracking stress [43].

### 3.4. Investigation of the Early-Age Stress Relaxation of Concrete Incorporated with MEA

In the fully constrained mode, the ideal elastic stress, which is calculated based on the early measured elastic modulus and free deformation, can be considered a significant factor for studying the stress relaxation of concrete.

(4) $$\sigma_{e}\left(t\right)=\sum \left[\varepsilon_{free}\left(t+∆t\right)-\varepsilon_{free}\left(t\right)\right]\times E\left(t\right)$$

where $\sigma_{e}\left(t\right)$ is the ideal elastic stress of the concrete at time *t*, in MPa; $\varepsilon_{free}\left(t\right)$ is the free deformation of concrete at time *t*, and the unit is $\mu \varepsilon ;E\left(t\right)\;$ is the elastic modulus of concrete at time *t*, in MPa.

Concrete is a kind of elastic–plastic material, and the magnitude of restraint stress in concrete at a specific moment reflects the combined effect of elastic stress and stress relaxation. Therefore, the relaxation coefficient of concrete, which is the ratio of actual restraint stress to ideal elastic stress, can be calculated as a measure of stress relaxation degree.

(5) $$K=\;\frac{\sigma }{\sigma_{e}}$$

where σ and $\sigma_{e}$ represent the restraint stress and ideal elastic stress, respectively, while *K* denotes the relaxation coefficient. The restraint stress of concrete can be measured directly by using the temperature stress testing machine, while the ideal elastic stress of concrete can be measured by combining the measured concrete deformation and the calculated elastic modulus.

When subjected to compression and tension, concrete exhibits compression creep and tensile creep, leading to distinct stress relaxation phenomena and necessitating different research formulas. In practical applications, concrete is often susceptible to cracks resulting from tensile forces. Therefore, in this study, we primarily investigated the stress relaxation behavior of concrete during the tensile stage.

**Figure 5:**
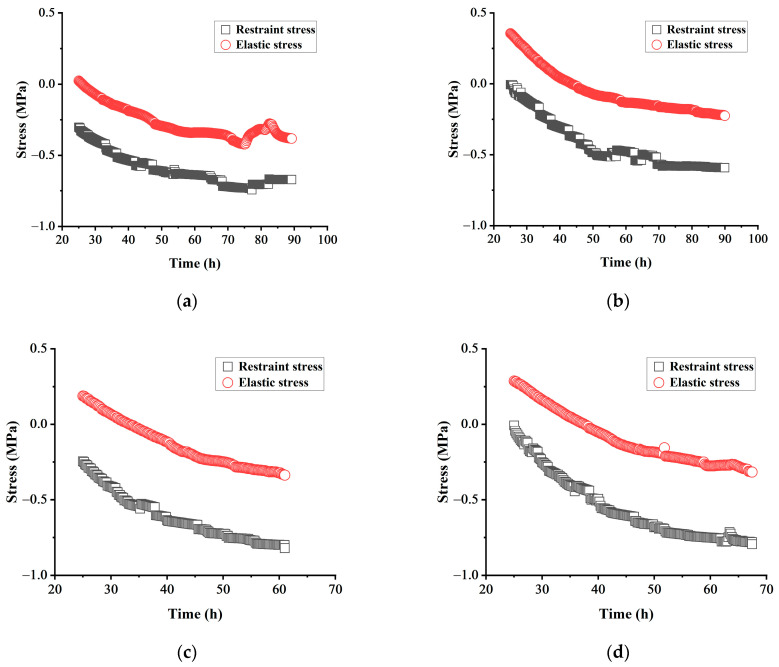
Comparison of restraint stress and elastic stress of each group: (**a**) comparison of restraint stress and elastic stress of H-1; (**b**) comparison of restraint stress and elastic stress of HM-1; (**c**) comparison of restraint stress and elastic stress of HM-2; (**d**) comparison of restraint stress and elastic stress of HM-3.

The specimens with four different mix proportions did not crack during the natural cooling stage, so the stress relaxation study was performed only up to the last sampling specimen point prior to initiating manual tension. Figure 5 shows a comparison of the restraint stress and the ideal elastic stress of various concrete test specimens. This figure shows that the relaxation of tensile stress in the test specimen begins after approximately 25 h.

Table 7 displays the comparison of expansion stress and cracking stress values for each group of specimens. Based on the observed changes in the relaxation coefficients of H-1 and HM-1 groups, it is evident that the stress relaxation level of concrete increases to a certain extent with an increase in MEA content. However, when the dosage of MEA continues to increase, there are no significant differences observed in the relaxation coefficients between HM-2, HM-3, and HM-1 groups. This is because, during the process of concrete hydration, MgO undergoes delayed hydration, leading to the gradual formation of Mg(OH)_2_ crystals within the cracks of cement crystals that have already formed. When the concrete is fully restrained, a certain number of minuscule cracks develop. These cracks are continually filled with newly generated Mg(OH)_2_ crystals [29], resulting in a continuous enhancement of the concrete’s stress relaxation level. However, due to the limited early-age hydration activity of MgO and the fixed water content in concrete, increasing the dosage of MEA beyond a certain proportion does not yield a significant improvement in the stress relaxation level of the concrete.

## 4. Development and Validation of the Predictive Model for Restraint Stress

The early-age restraint stress of concrete is influenced by concrete deformation and elastic modulus, taking into account stress relaxation. The parameters needed for calculating early-age restraint stress are as follows:

1. Thermal expansion coefficient;
2. Autogenous shrinkage;
3. Modulus of elasticity;
4. Relaxation coefficient.

The calculation of the thermal expansion coefficient has already been explained in the previous section. Therefore, in this section, we focus on determining the mathematical models for autogenous shrinkage, elastic modulus, and relaxation coefficient.

### 4.1. Selection of Early Autogenous Shrinkage Model for Concrete Mixed with MEA

Currently, numerous scholars and teams have proposed autogenous shrinkage models for early-age concrete. In this study, the CEB-FIP [44], EN-1992 [45], Tazawa [46], and RILEM [47] autogenous shrinkage models were selected for comparison with the measured data. The most appropriate autogenous shrinkage model was selected. The specific formulas are presented in Table 8.

**Figure 6:**
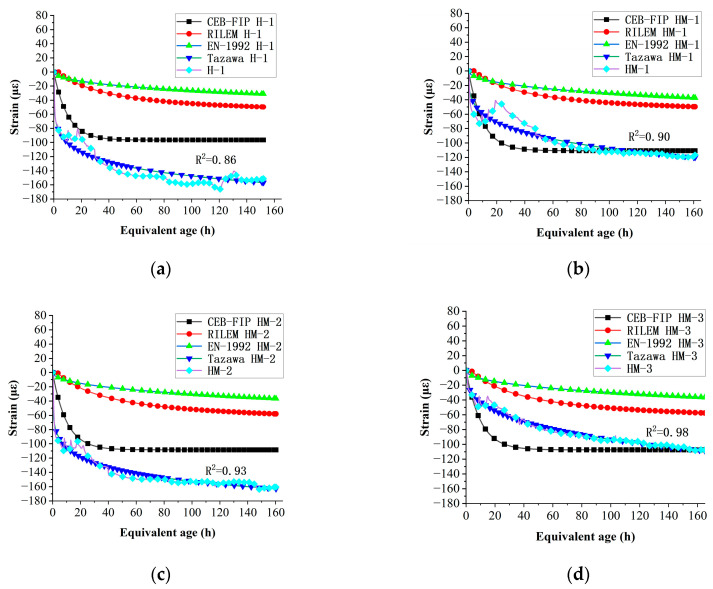
Comparison of autogenous shrinkage model of each group: (**a**) comparison of autogenous shrinkage model of H-1; (**b**) comparison of autogenous shrinkage model of HM-1; (**c**) comparison of autogenous shrinkage model of HM-2; (**d**) comparison of autogenous shrinkage model of HM-3.

The comparison between the final calculated model data and the measured data is shown in Figure 6.

In this experiment, the determination coefficient *R*^2^ was used as the evaluation index. The closer *R*^2^ is to 1, the higher the accuracy is. The expression is as follows:

(6) $$R^{2}=1-\frac{{SS}_{eer}}{{SS}_{tot}}$$

(7) $${SS}_{eer}=\sum {\left(y_{i}-f_{i}\right)}^{2}$$

(8) $${SS}_{tot}=\sum {\left(y_{i}-\bar{y}\right)}^{2}$$

In the above formula, $R^{2}$ is the coefficient of determination, ${SS}_{eer}$ and ${SS}_{tot}\;represent$ the residual sum of squares and the total sum of squares. $y_{i}$ represents the predicted value, $f_{i}$ is the test value, and $\bar{y}$ is the average value of test values.

It can be seen from the comparison between the four models and the measured data that the calculation results of the Tazawa model are closest to the actual autogenous shrinkage value.

It can be seen from the comparison between the four models and the measured data that the calculation results of the Tazawa model are closest to the actual autogenous shrinkage value. The Tazawa model yielded determination coefficients of 0.86, 0.90, 0.93, and 0.98 for H-1, HM-1, HM-2, and HM-3, demonstrating good performance and suitability for MEA concrete mixture design.

### 4.2. Investigation of the Early-Age Elastic Modulus of Concrete Incorporated with MEA

At present, most codes and research groups believe that the elastic modulus of concrete primarily depends on the composition of the paste and aggregate. Specifically, the volume ratio and the strength of the interfacial transition zone between the aggregate and the cement matrix significantly influence the elastic modulus of concrete. In this study, two time-dependent elastic modulus development models were used to compare with the elastic modulus measured with TSTM.

1. Lin Zhi Hai elastic modulus model [5]:

(9) $$E_{C}\left(t\right)=\frac{E_{1}}{1+{\left(\frac{t_{c1}}{t}\right)}^{a_{1}}}+\frac{E_{2}}{1+{\left(\frac{t_{c2}}{t}\right)}^{a_{2}}}+\frac{E_{3}}{1+{\left(\frac{t_{c3}}{t}\right)}^{a_{3}}}$$

(10) $$t_{c1}=t_{c0}-{\left(\frac{∆T}{30}\right)}^{\frac{1}{3}}+{\left[20\times \left(\frac{W}{B}-0.45\right)\right]}^{\frac{1}{3}}$$

In the above formula, *E*_1_ is the concrete’s elastic modulus of 24 h, and the unit is GPa;

*E*_2_ is the increase in elastic modulus of the concrete at 7 days, in GPa;

*E*_3_ is the increase in elastic modulus of the concrete at 28 days, in GPa;

*t_c_*_0_ is the time when *E*_1_ develops to half, and the unit is h;

*t_c_*_1_ is the value of *t_c_*_0_ corrected in consideration of the temperature rise at an early age and the water–binder ratio, in h;

*t_c_*_2_ is the time when *E*_2_ develops to half, and the unit is h;

*t_c_*_3_ is the time when *E*_3_ develops to half, and the unit is h;

Δ*T* is the maximum temperature rise, in °C;

a_1_, a_2_, and a_3_ is a constant.

The coefficients of the four mix proportions are shown in Table 9.

2. CEB/FIP MC90 [48]:

(11) $$E_{c}=E_{28}\times {\left\{exp\left[s\times \left(1-\sqrt{\frac{28-t_{0}}{t-t_{0}}}\right)\right]\right\}}^{0.5}$$

where *E*_28_ is the elastic modulus of the concrete at 28 days, in GPa; *s* is the parameter of hydration development, for rapid hardening and high-strength cement, which is 0.2, and for ordinary cement, it is 0.25; *t*_0_ is the initial setting time in h, which was set as 8 h in this study.

**Figure 7:**
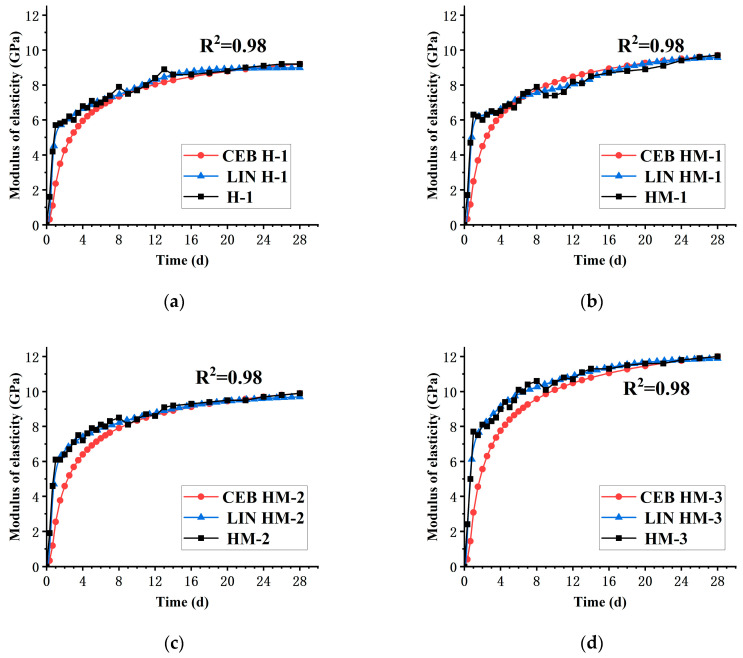
Comparison of elastic modulus development of each group: (**a**) comparison of elastic modulus development of H-1; (**b**) comparison of elastic modulus development of HM-1; (**c**) comparison of elastic modulus development of HM-2; (**d**) comparison of elastic modulus development of HM-3.

The comparison between the theoretical value calculated with the two models and the measured value is shown in Figure 7.

It can be seen from the comparison curves between the measured results of the elastic modulus of the four groups of mix proportions and the calculated results of the model that the Lin Zhi Hai model can better predict the development of the elastic modulus of concrete under the temperature stress test. The elastic modulus of H-1, HM-1, HM-2. and HM-3 were calculated with the Lin Zhi Hai model, and the determination coefficient values were 0.98, 0.98, 0.98, and 0.99, respectively.

### 4.3. Validation of the Predictive Model for Restraint Stress

Regression analysis was performed on the data presented in Section 3.4 to derive the formula for the relaxation coefficient, which is shown below:

(12) $$K\left(t\right)=5.43599-\frac{3789.83207}{\left(1+t^{1.78063}\right)}$$

where *t* is the age of the concrete, and the unit is h.

Once the expressions for concrete deformation, elastic modulus, and stress relaxation coefficient are determined, it is possible to establish the expression for the time-dependent development of concrete restraint stress.

(13) $$\sigma =\int \left(\alpha_{T}\cdot dT/dt+d\varepsilon_{c}\left(t\right)/dt\right)\cdot E\left(t\right)\cdot K\left(t\right)dt$$

**Figure 8:**
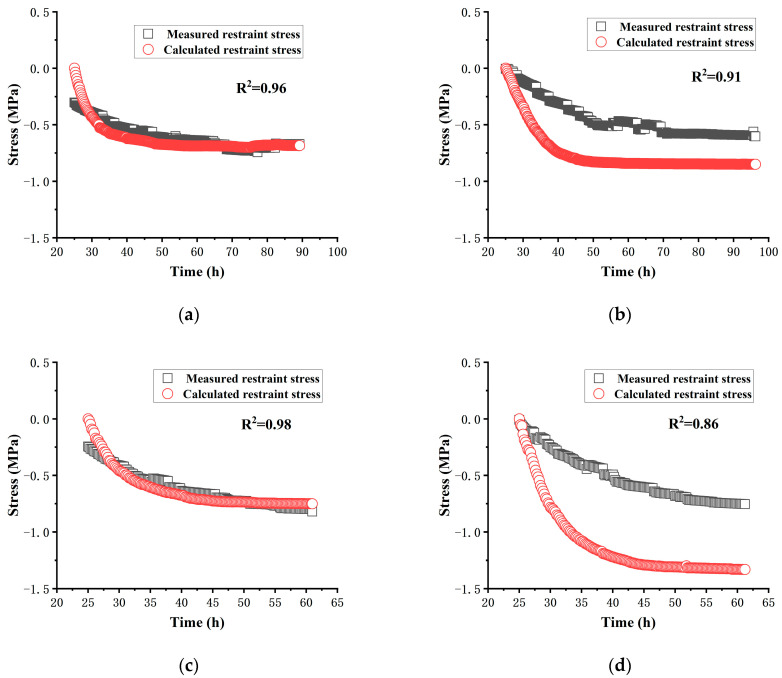
Comparison of calculated and measured restraint stress of each group: (**a**) comparison of calculated and measured restraint stress of H-1; (**b**) comparison of calculated and measured restraint stress of HM-1; (**c**) comparison of calculated and measured restraint stress of HM-2; (**d**) comparison of calculated and measured restraint stress of HM-3.

The Figure 8 below illustrate the comparison between the restraint stress calculated using the established concrete stress prediction model and the measured restraint stress.

From the data in the figure, it can be seen that overall, the model can better predict the tensile restraint stress of concrete after 25 h.

## 5. Conclusions

In this study, we performed free deformation and full constraint tests on concrete samples with various MEA admixtures using TSTM. We also analyzed the deformation curves and stress curves. Thus, the following conclusions were drawn:

1. Temperature stress tests were performed on four types of concrete mix proportions, ranging from free deformation tests to fully restrained tests. Based on the experiments, the deformation and mechanical properties of concrete with different MEA admixtures were investigated. The experimental data indicate that with the continuous increase in MEA dosage, the temperature expansion coefficient, the autogenous shrinkage value, and the free deformation value of concrete exhibit a trend of initially increasing and then decreasing. The peak expansion stress and cracking stress gradually increase, while the stress relaxation level continues to increase, with a maximum increase of 65.5%. However, when the MEA dosage exceeds 5%, there is no significant increase in stress relaxation.

Based on this analysis, the addition of the MEA expansion agent appears to exhibit a certain inhibitory effect on concrete cracking. Nevertheless, exceeding a certain threshold dosage of MEA results in a reduced impact of this inhibitory effect. This provides a reference for determining the optimal amount of MEA that can be added to enhance the mechanical properties of concrete and achieve a more pronounced effect.

Currently, there are two primary concerns regarding temperature stress testing. Firstly, there is a lack of standardized testing methods for temperature stress testing; thus, further investigation into testing techniques is required. Secondly, because the principle of temperature stress testing involves uniaxial constraints, there exist discrepancies between the testing conditions and practical engineering applications. The crack resistance suggestions obtained through temperature stress testing must be further validated in actual engineering applications.

2. The comparison of the restraint stress model results with the measured restraint stress demonstrates that the early model can predict the restraint stress during the tensile stress relaxation phenomenon after 25 h.

This prediction model has certain limitations. Firstly, although the process and operation methods were the same during the free deformation and fully constraint experiments conducted with TSTM in this study, there were differences in the temperature history of the concrete under the two modes, resulting in certain errors between the calculated theoretical stress and the actual stress, which affected the experimental accuracy. Secondly, this study proposes a model that addresses the stress relaxation phenomenon caused by tensile creep after 25 h but does not consider the stress relaxation phenomenon caused by the compressive creep of concrete and its subsequent impact. This is because stress relaxation mechanisms generated due to the compressive creep and tensile creep differ, requiring different empirical formulas. Additionally, early deformation and changes in mechanical properties are complex, necessitating more data and accurate models to consider the influence of different concrete materials and other factors on temperature.

## Figures and Tables

**Table 1:** Cement performance indicators.

Quality Index											
Performance Index	Setting Time		Compressive Strength /MPa		Flexural Strength /MPa		MgO (%)	SO_3_ (%)	Cl^−^ (%)	Loss on Ignition (%)	Bulk Density t/m^3^
	Initial Setting Time (min)	Final Setting Time (min)	3d	28d	3d	28d					
PO42.5	172	234	27.2	51.7	5.5	8.2	3.71	2.51	0.043	4	1.3
GB175-2007	≥45	≤600	≥17	≥42.5	≥3.5	≥6.5	≤5.0	≤3.5	≤0.06	≤5.0	—

**Table 2:** Sieving table for coarse aggregate.

Coarse Aggregate Type (mm)	Content (%)					
	2.5~5 mm	5~10 mm	10~15 mm	15~20 mm	20~25 mm	Total
5~10	7.5	92.5	0	0	0	100
10~20	0	0	77.8	22.2	0	100

**Table 3:** Performance indicators of MEA.

MEA	Technical Requirement					
	Setting Time		Limit Expansion Rate/%		Compressive Strength/MPa	
	Initial Setting Time/min	Final Setting Time/min	7d in Water	21d in the Air	7d	28d
Experimental results	206	256	0.040	−0.014	35.2	45.3
Standard requirements	≥45	≤600	≥0.035	≥−0.015	≥22.5	≥42.5

**Table 4:** Mix proportion of concrete.

Group No	Water–Binder Ratio	Water	Cement	Fly Ash	MgO	Coarse Aggregate	Fine Aggregate	Sand Percentage	Water Reducing Agent
		(kg)	(kg)	(kg)	(kg)	(kg)	(kg)	(%)	(kg)
H-1	0.34	182	352	186	0	892	805	47.4	1.60
HM-1	0.34	182	352	186	16.13	892	805	47.4	1.66
HM-2	0.34	182	352	186	26.88	892	805	47.4	1.70
HM-3	0.34	182	352	186	43.00	892	805	47.4	1.74

**Table 5:** The thermal expansion coefficient of each group of specimens.

Group No	Thermal Expansion Coefficient
H-1	3.36
HM-1	4.21
HM-2	4.17
HM-3	3.61

**Table 6:** The peak expansion stress and cracking stress values of each specimen group.

Group No	Peak Value of Expansion Stress	Cracking Stress
H-1	0.12	−0.67
HM-1	0.18	−1.20
HM-2	0.24	−1.28
HM-3	0.32	−1.31

**Table 7:** The peak expansion stress and cracking stress values of each specimen group.

Group No	Maximum Tensile Stress	Corresponding Elastic Stress	Relaxation Coefficient
H-1	0.67	0.38	1.76
HM-1	0.60	0.24	2.50
HM-2	0.82	0.34	2.41
HM-3	0.80	0.32	2.50

**Table 8:** Summary of concrete autogenous shrinkage models.

Autogenous Shrinkage Model	Parameter Description
CEB-FIP [44]: $\varepsilon_{cas}\left(t\right)=\varepsilon_{ca0}\left(f_{cm}\right)\beta_{as}\left(t\right)$$\varepsilon_{ca0}\left(f_{cm}\right)=-\alpha_{as}{\left(\frac{\frac{f_{cm}}{f_{cm0}}}{6+\frac{f_{cm}}{f_{cm0}}}\right)}^{2.5}$$\beta_{as}\left(t\right)=1-exp\left(-0.2\sqrt{\frac{t}{t_{1}}}\right)$	$\;\varepsilon_{cas}\left(t\right)$ represents the autogenous shrinkage of the concrete at the moment t (με), $f_{cm}$$\;\mathrm{is}\;\mathrm{the}\;\mathrm{compressive}\;\mathrm{strength}\;\mathrm{of}\;\mathrm{the}\;\mathrm{concrete}\;\mathrm{cube}\;\mathrm{at}\;28\;\mathrm{days}\;(\mathrm{MPa}),\;f_{cm}=f_{ck}+∆f$, $∆f=8\;MPa,\;f_{ck}$$\;\mathrm{is}\;\mathrm{the}\;\mathrm{compressive}\;\mathrm{strength}\;\mathrm{of}\;\mathrm{the}\;\mathrm{concrete}\;\mathrm{cylinder}\;\mathrm{at}\;28\;\mathrm{days}\;(\mathrm{MPa}),\;f_{cm0}=10MPa,\alpha_{as}$$\;\mathrm{is}\;\mathrm{the}\;\mathrm{parameter}\;\mathrm{for}\;\mathrm{the}\;\mathrm{type}\;\mathrm{of}\;\mathrm{cement}\;(700\;\mathrm{for}\;\mathrm{plain}\;\mathrm{concrete}),\;\mathrm{and}\;t_{1}$ is 1d and *t* is the age of the concrete (d).
EN-1992 [45]: $\varepsilon_{ca}\left(t\right)=\beta_{as}\left(t\right)\cdot \varepsilon_{ca}\left(\infty \right)$$\varepsilon_{ca}\left(\infty \right)=-2.5\cdot \left(f_{ck}-10\right)$$\beta_{as}\left(t\right)=1-exp\left(-0.2t^{0.5}\right)$	$\varepsilon_{ca}\left(t\right)$ represents the autogenous shrinkage of the concrete at the moment $\left(\mu \varepsilon \right)$$,\;\beta_{as}\left(t\right)$$\;\mathrm{is}\;\mathrm{the}\;\mathrm{self}-\mathrm{shrinking}\;\mathrm{parameter},\;\varepsilon_{ca}\left(\infty \right)$ is the self-shrinking final value (με), $f_{ck}$ is the compressive strength of the concrete cylinder at 28 days (MPa), and *t* is the age of the concrete (d).
Tazawa [46]: $\varepsilon_{c}\left(t\right)=\varepsilon_{co}\left(w/c\right)\beta_{a}\left(t\right)$$\beta_{a}\left(t\right)=1-exp\left\{-a{\left(t-t_{0}\right)}^{b}\right\}$$\varepsilon_{co}\left(w/c\right)=-3070\times exp\left\{-7.2\times \left(w/c\right)\right\}$	$\varepsilon_{c}\left(t\right)$ represents the autogenous shrinkage of the concrete at time t (με$),\;\varepsilon_{co}\left(w/c\right)$ for the final value of concrete autogenous shrinkage (με), a and b$\;\mathrm{is}\;a\;\mathrm{constant},\;t_{0}$ is the pouring time of concrete (h), and *t* is the age of concrete (h).
RILEM [47]: $\frac{f_{c}\left(t\right)}{f_{c28}}<0.1$; $\varepsilon_{cas}\left(t,f_{c28}\right)=0$ $\frac{f_{c}\left(t\right)}{f_{c28}}\;\geq \;0.1$; $\varepsilon_{cas}\left(t,f_{c28}\right)=-\left(f_{c28}-20\right)\times \left(\frac{2.2f_{c}\left(t\right)}{f_{c28}}-0.2\right)$	$\varepsilon_{cas}\left(t,f_{c28}\right)$ represents the autogenous shrinkage of the concrete at time t(με), *t*$\;\mathrm{is}\;\mathrm{the}\;\mathrm{age}\;\mathrm{of}\;\mathrm{concrete}\;(d),\;f_{c28}$$\;\mathrm{is}\;\mathrm{the}\;\mathrm{compressive}\;\mathrm{strength}\;\mathrm{of}\;\mathrm{the}\;\mathrm{concrete}\;\mathrm{cylinder}\;\mathrm{at}\;28\;\mathrm{days}\;(\mathrm{MPa}),\;f_{c28}=f_{ck}+∆f$, ∆f=8 MPa$,\;f_{ck}$$\;\mathrm{is}\;\mathrm{the}\;\mathrm{compressive}\;\mathrm{strength}\;\mathrm{of}\;\mathrm{the}\;\mathrm{concrete}\;\mathrm{cube}\;\mathrm{at}\;28\;\mathrm{days}\;(\mathrm{MPa}),\;\mathrm{and}\;f_{c}\left(t\right)$ is the compressive strength of the concrete cylinder at time *t* (MPa).

**Table 9:** Elastic modulus parameters of the Lin Zhi Hai model.

Group No	*E*_1_	*E*_2_	*E*_3_	*t_c_*_1_	*t_c_*_2_	*t_c_*_3_	a _1_	a _2_	a _3_	∆T
	(GPa)	(GPa)	(GPa)	(h)	(h)	(h)				(°C)
H-1	5.7	1.7	1.6	13.9	90	270	3.89	2.96	5.31	14.5
HM-1	6.3	1.3	2.1	13.8	120	384	3.93	5.08	4.73	19.3
HM-2	6.1	2.2	1.6	13.8	80	312	3.07	1.75	2.92	19.9
HM-3	7.7	2.7	1.6	12.1	90	324	2.75	2.60	3.69	17.3

## Data Availability

Data are contained within the article.
